# Patient with Crouzon Syndrome Treated with Modified Le Fort III Osteotomy without Previous Orthodontic Treatment: Case Report and a Review of the Literature

**DOI:** 10.1155/2020/6248971

**Published:** 2020-01-20

**Authors:** Farnoosh Mohammadi, Afrooz Javanmard, Hamid Mojtahedi

**Affiliations:** ^1^Craniomaxillofacial Research Center, Oral and Maxillofacial Surgery Department, Shariati Hospital, Tehran University of Medical Sciences, Tehran, Iran; ^2^Department of Prosthodontics and Dental Research Center, Faculty of Dentistry, Tehran University of Medical Sciences, Tehran, Iran

## Abstract

Crouzon syndrome is the most common type of craniofacial dysostosis anomaly which presents a great challenge for clinicians since birth. Multiple synostoses in the sutures of the cranial base in this syndrome result in the hypoplasia of the midface, shallow orbits, a short nasal dorsum, maxillary hypoplasia, and, in severe cases, obstruction of the upper airways. Apart from esthetic and functional problems, these patients suffer from various psychological problems which mandate correction of midface deformities at younger ages. The aim of this report is to describe the case of a 26-year-old female patient with Crouzon syndrome displaying severe midface hypoplasia and proptosis with no history of orthodontic treatment, who was treated with modified Le Fort III osteotomy with a coronal and intraoral approach without periocular incisions.

## 1. Introduction

In 2012, Crouzon described the triad of skeletal deformity, facial anomalies, and exophthalmos as hereditary craniofacial dysostosis [1]. Crouzon syndrome is known as an autosomal dominant disorder with complete penetration and varying expression [2]. The prevalence of this syndrome is approximately 1 : 25000 births [3]. Premature closure of the coronal and sagittal sutures of the cranium in this syndrome results in the abnormal growth of the skull base, orbits, and maxillary complex [4]. Despite other autosomal dominant craniosynostosis syndromes, no digital anomalies are seen in the Crouzon syndrome [5]. Patients with Crouzon syndrome have normal mental development. However, in a small number of cases, there is a delay in cognitive development, which might be attributed to an increase in intracranial pressure during infancy [6].

Crouzon syndrome is due to a mutation in the FGFR-2 (fibroblast growth factor receptor-2) gene. It has been reported that in 50% of cases, Crouzon syndrome is due to a new mutation without a hereditary pattern [7]. Multiple synostoses in the sutures of the cranial base in this syndrome result in the hypoplasia of the midface, shallow orbits, a short nasal dorsum, maxillary hypoplasia, and, in severe cases, obstruction of the upper airways [8]. The manifestations of Crouzon syndrome during infancy might vary from minor characteristics in association with minor defects in the midface to the involvement of several sutures in the skull and severe midface deficiency and visual problems [9]. An increase in intracranial pressure might result in optic nerve atrophy and loss of vision if no intervention is made [10]. These patients might have other concomitant skeletal problems such as mandibular prognathism and/or retrognathism, possibly in association with midface deformity [11].

Apart from systemic and dental problems, patients with craniofacial synostosis suffer from various psychological problems due to defects in their appearance. As a result, these patients have more limited social activities and problems with communication with their peers [12]. Correction of midface deformities at younger ages might have a significant role in decreasing the psychological problems of these patients [13]. The important role of the face in vision, respiration, speech, smelling, and hearing makes the defects that affect this area more important than the esthetic problems [11].

The aim of this report is to describe the case of a 26-year-old female patient with Crouzon syndrome displaying severe midface hypoplasia and proptosis with no history of orthodontic treatment, who was treated with modified Le Fort III osteotomy with a coronal and intraoral approach without periocular incisions.

## 2. Diagnosis and Patient History

**Figure 1:**
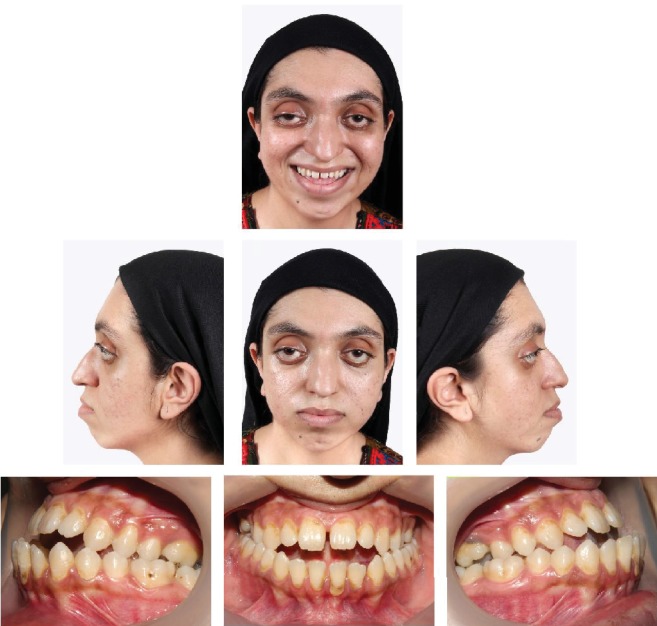
Pretreatment patient's extraoral and intraoral photographs.

**Figure 2:**
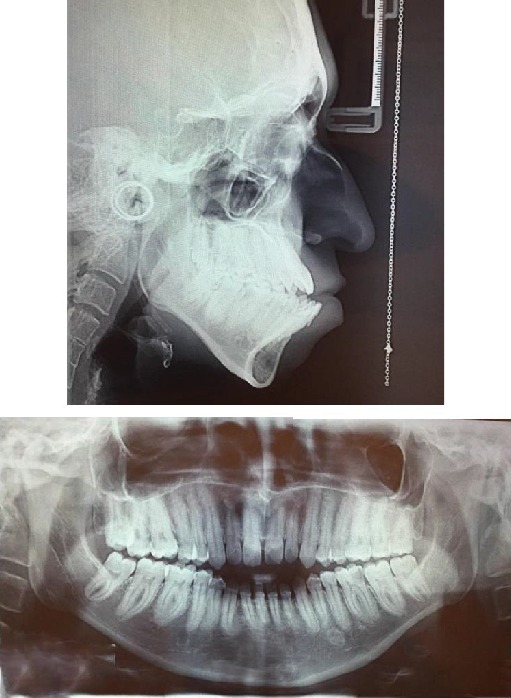
Pretreatment lateral cephalogram and panoramic views.

**Figure 3:**
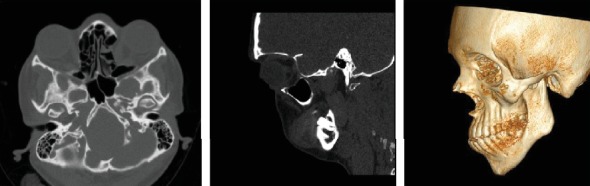
Pretreatment axial, sagittal, and three-dimensional computed tomographic views.

The case described here is a 26-year-old female patient who referred to the Shariati Medical Center of Tehran, Iran, for treatment at this age. The chief complaint of the patient was facial abnormality and the inability to close the eyes. Clinical examinations revealed severe defects in the lower orbit area and the midface, moderate hypertelorism, and severe exophthalmos (Figure 1). The patient did not exhibit any signs of an increase in intracranial pressure and neurologic problems and had normal mental development. Consultation with an ophthalmologist revealed an approximately 30% decrease in visual acuity and amblyopia. Funduscopic examinations of the eye did not reveal any signs indicating papilledema. The patient did not complain of nocturnal apnea and respiratory obstruction. Intraoral examinations revealed a 5 mm anterior open bite and bilateral posterior crossbite. The molar and canine relationships were Cl III on both sides, and a reverse overjet of approximately 6 mm was measured (Figure 1). In cephalometric evaluations, she had retrusive maxilla with SNA angle of 80 (Table 1 and Figure 2). There were no signs of crowding, and the maxillary and mandibular teeth had diastema. The amount of tooth show at rest and smiling had decreased, and paranasal deficiency was evident. Examinations showed that the upper and lower extremities were intact and there were no digital deformities. After the evaluations, a diagnosis of Crouzon syndrome was achieved. Owing to financial and social problems, the patient had not referred for treatment at younger ages and was not interested in orthodontic treatment in order to correct the dental status. Clinical evaluations showed proper contour of the forehead. The nasofrontal area had a proper projection, and the length of the nose was normal. The scleral show had increased, and the dystopia of the lateral canthus was seen. The facial axial CT scan views revealed shallow orbits on both sides and exorbitism (Figure 3). The patient exhibited no cleft palate and velopharyngeal insufficiency; however, the malocclusion and anterior open bite had resulted in speech problems.

## 3. Treatment Objectives

Given the patient's chief complaint and her refusal to undergo orthodontic treatment due to financial problems, the aim of treatment was to correct the hypoplasia of the midface and achieve a proper occlusal relationship. Due to minor hypertelorism, its correction was not included in the treatment plan. The objectives of the treatment in this patient were as follows based on the problems described above: (1) correction of the midface and infraorbital defects with midface advancement; (2) decreasing ocular proptosis; (3) no change in the nasal projection and contour of the forehead, considering their normality; (4) increasing maxillary tooth show at rest and smiling; and (5) closing the open bite and achieving maximum intercuspation at occlusion in the absence of orthodontic treatment.

## 4. Surgical Options for the Correction of Midface Deficiencies

The treatment options in patients with craniofacial dysostosis are very divergent, depending on the severity of skeletal and dental problems and the patients' psychosocial status. Facial skeletal deformities affect different structures in the form of repeated patterns; however, their expression and the severity of involvement are variable at different syndromic and nonsyndromic cases, affecting the treatment plans and the surgical correction of deformities [14]. Treatment of the sagittal midface deficiency in these patients consists of routine Le Fort osteotomies with or without distraction osteogenesis. The currently used osteotomy techniques to correct the midface deformities in patients with craniofacial dysostosis are generally divided into four categories:

1. *Subcranial Monoblock Advancement Osteotomy*. This is used to correct the general midface defects. It is the treatment of choice in cases in which the infraorbital, supraorbital, and the nasomaxillary complexes have a total defect
2. *Le Fort III Extracranial Osteotomy*. This is a proper treatment option in cases in which the frontal and supraorbital areas are normal and the defect is located in the middle third of the face, affecting the maxilla, infraorbital rim, and nasal unit
3. *Modified Le Fort III Osteotomy*. This is used in the treatment of midface defects when the nasomaxillary complex is not involved
4. *Le Fort II Osteotomy*. This treatment option is used when the extent of involvement of the orbital and zygomatic areas is less than that of the nasomaxillary areas (David, 2015).

Based on the results of clinical examinations and radiographic evaluations in the present study, which indicated the absence of any defects in the upper third of the face and the normal contour of supraorbital and frontal areas, monoblock advancement was not a proper choice in this patient. On the other hand, the patient did not exhibit nasomaxillary hypoplasia, and the classic Le Fort III osteotomy could be associated with excessive advancement of the nasal unit, which is esthetically inappropriate. Considering what was discussed above and the defect in the malar and maxillary areas, the anterior open bite, and the absence of proper dental arch, modified Le Fort III osteotomy in association with mandibular osteotomy after initial orthodontic treatment was suggested to the patient. The patient refused to undergo presurgical orthodontic treatment due to social and financial problems. Therefore, the modified Le Fort III osteotomy was decided, without orthodontic treatment, to correct the occlusion, the midface deficiency, and the exophthalmos.

Treatment of skeletal deformities and the midface defects has long been a great challenge for surgeons. In 1901, Le Fort and Tessier classified the different patterns of facial skeletal fractures for the first time [15]. Gillies and Harrison were the first to attempt to treat the facial defects of patients with craniofacial dysostosis by moving the midface in 1950. The advancement of the midface in association with external fixation did not result in favorable long-term stability [16]. Finally, in 1957, the efforts made by Tessier revolutionized the surgical treatment of the general midface defects by introducing intracranial and extracranial access to mobilize the midface. Tessier achieved stable long-term results in patients with craniofacial dysostosis by internal rigid fixation for the first time. Development of surgical techniques and the introduction of various modifications by Tessier and other surgeons resulted in improvements in the functional defects and facial appearance of patients with midface deficiencies [17].

## 5. Treatment

**Figure 4:**
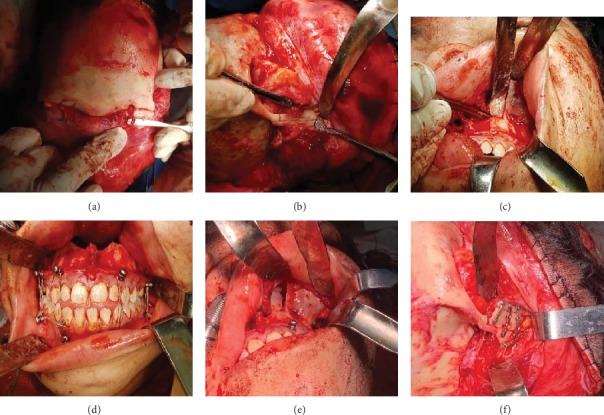
(a) The supraorbital nerve was released by a fissure bur and an osteotome and preserved above the eye globe afterward. (b) The vertical osteotomy line was initiated midway in this area and continued up to the lowest limit of the zygomatic buttress. (c) The osteotomy line which was extended vertically from the infraorbital rim from the relative site of orbital floor osteotomy termination up to the nasal cavity lower rim level and pyriform aperture. (d) The maxillary teeth were fixed in an edge-to-edge position with the mandibular teeth by IMF screws. (e) Amount of gap in the pyriform aperture area after disimpaction and advancement. (f) Rigid fixation was carried out in lateral orbital rim and zygomatic buttress areas by titanium microplates.

First, nasotracheal intubation was carried out by placing the tube inferiorly and moving it through the cheek. Oral intubation is not suggested in such cases because it is necessary to achieve occlusion and intermaxillary fixation during the surgical procedure. The tracheal tube was fixed to the membranous septum and columella, using sutures. Bilateral tarsorrhaphy suturing was carried out in order to protect the eye globe and the cornea. The coronal approach in association with intraoral approach was considered without access to periocular structures to make sure of the direct access to modified Le Fort III osteotomy field. Before draping the patient, the hairline was determined at a distance of 2 cm, and the hair anterior and posterior to the incision line was braided. 2% lidocaine with 1 : 100000 epinephrine was injected into the coronal incision line and the maxillary vestibular mucosa. Coronal approach was performed with a cutaneous incision in the midauricular area on one side ending on the midauricular area on the other side crossing the superior temporal line, approximately 2 cm behind the hairline on both sides. The cutaneous incision carried out to the depth of the galea layer on the pericranium, and then the supraperiosteal dissection in the avascular tissue plane continued up to approximately 2 cm above the superior rim of the orbit. In this area, a sharp incision was made using a scalpel on the pericranium, and the periosteum of the frontal area was elevated to gain full access to the upper rim and the lateral rim of the orbit. Electrocautery was used, and the coronal flap margins were sutured with 2-0 silk sutures to achieve hemostasis. The temporalis muscle was completely elevated in the subperiosteal plan superiorly from the superior temporal line on both sides up to the zygomatic arch area in order to gain access to the buttress and zygomatic arch. After completely releasing the periosteum from the superior orbital rim, the supraorbital nerve was released by a fissure bur and an osteotome and preserved above the eye globe afterward (Figure 4(a)). Next, complete periorbital dissection was carried out up to the infraorbital fissure from the lateral aspect, preserving the adhesion of the medial canthus. Dissection of 3/4 of the globe circumference prepared the lateral and inferior rims and the floor of the orbit for osteotomy. A proper dissection was carried out in the subperiosteal plane to make sure of preservation of the facial nerve. Subsequently, access was provided to the piriform rim, zygomatic buttress, and pterygomaxillary areas on both sides by extending the circumvestibular incision between the first molar areas on both sides up to the midline. Keeping the dissection in the subperiosteal plane preserves the important arteries in this area, including the internal maxillary artery. After making sure of the complete release of the infraorbital nerve, the nasal cavity floor, and the anterior area of the nasal septum, dissection was terminated, and osteotomy was instituted.

First, the frontozygomatic suture was identified, and the osteotomy was initiated from the area superior to the suture with the use of a saw. Before terminating the transverse osteotomy line, the vertical osteotomy line was initiated midway in this area and continued up to the lowest limit of the zygomatic buttress (Figure 4(b)). Consideration should be given through the preservation of the globe soft tissue by malleable retractors, and the osteotomy of the lateral rim of the orbit continued from 1 cm posterior to the rim up to the infraorbital tissue with the use of a 2 mm osteotome. The bone at the floor of the orbit is paper-thin and can be osteotomized easily and with greater control, using an osteotome with no need for a saw and a fissure bur. Once the osteotomy of the infraorbital fissure and orbital floor had terminated, the Langenbeck retractor was fixed at the infraorbital rim and medial to infraorbital foramen through intraoral access, providing adequate space for osteotomy. The osteotomy line was extended vertically from the infraorbital rim from the relative site of orbital floor osteotomy termination up to the nasal cavity lower rim level (Figure 4(c)). Then, the direction of osteotomy was changed to continue horizontally to terminate at the piriform rim. Finally, similar to Le Fort I osteotomy, the vomer and nasal septum were completely tapped with the use of a nasal septum osteotome. To make sure of the osteotomy of the posterior wall of the maxilla and the pterygomaxillary area, the osteotomy of the pterygoid plates was completed through both extraoral access behind the zygomatic arch and intraoral access behind the maxillary tuberosity. Once we made sure that the osteotomy had been perfectly done, row forceps were placed at the nasal cavity floor and the hard palate, and disimpaction was initiated. After that, the midface is relatively released; row forceps were used to increase the range of midface mobilization. It is of paramount importance to make sure of complete mobility of the midface in order to achieve long-term and stable results. After that, the midface was advanced with the use of intermaxillary fixation screws which were placed at 8 points in the mandible, and maxilla and the maxillary teeth were fixed in an edge-to-edge position with the mandibular teeth (Figure 4(d)). The amount of exophthalmos correction, advancement of the inferior orbital rim, and projection of the malar and paranasal areas were evaluated (Figure 4(e)). The rigid fixation process was instituted with a gap of almost one cm in the frontozygomatic suture and one cm of advancement of the midface. Then, cortical monoblock bone graft was harvested paramedially from the parietal bone of one side with the use of a fissure bur and an osteotome. The harvested bone grafts were placed at frontozygomatic osteotomy site gaps in order to preserve the continuity of bone and decrease the chance of skeletal relapse. Subsequently, rigid fixation was carried out in lateral orbital rim and zygomatic buttress areas by microplates (Figure 4(f)). The tarsorrhaphy sutures were released on both sides, and force duction test was performed to make sure of the absence of any entrapment on the floor of the orbit and complete movement of the eye globe is restored. The temporalis muscle was suspended to the pericranium of the sculp in the overcorrected position, and hemostasis was ensured. Surgical field was debrided with copious irrigation, and the intraoral incision was sutured with 4-0 vicryl sutures; the coronal surface was sutured with 3-0 vicryl and 2-0 nylon sutures in two layers. A pressure pack was placed in the scalp area in order to redrape the soft tissue and to decrease the postoperative edema. The patient was extubated at the end of surgery for maintaining the airway condition; there was no reason to extend the patient's intubation period. Steroids and cephalosporins were prescribed before surgery and repeated every 4 hours during surgery. The steroids continued for 48 hours, and oral antibiotics were given for 10 days after surgery. In such surgeries, early ambulation and oral nutrition should be encouraged. Decongestant sprays were used for two weeks. A soft diet was continued for six weeks. Elastic guides were delivered to the patient for reducing the patient discomfort and control of occlusion.

## 6. Discussion

It is a challenge to treat patients with craniofacial dysostosis syndromes. Craniofacial teams consist of several specialists, including ophthalmologists, neurosurgeons, oral and maxillofacial surgeons, plastic surgeons, and orthodontists, who should contribute since birth in the diagnosis and treatment of these patients. The reconstruction in these patients should be carried out in multiple stages in order to accommodate the facial and cerebral growth patterns and the patients' cognitive and psychological statues. The patient described in this report was different from the routine treatment stages of such patients in many ways. The patient was from a family with low socioeconomic status and lived in one of the border towns in Iran. She had not undergone routine treatments at younger ages and did not have the required financial potential to accept orthodontic treatment. Preoperative orthodontic alignment of the teeth could have provided adequate space for correcting the occlusion in one step during the surgery in association with mandibular osteotomy.

**Figure 5:**
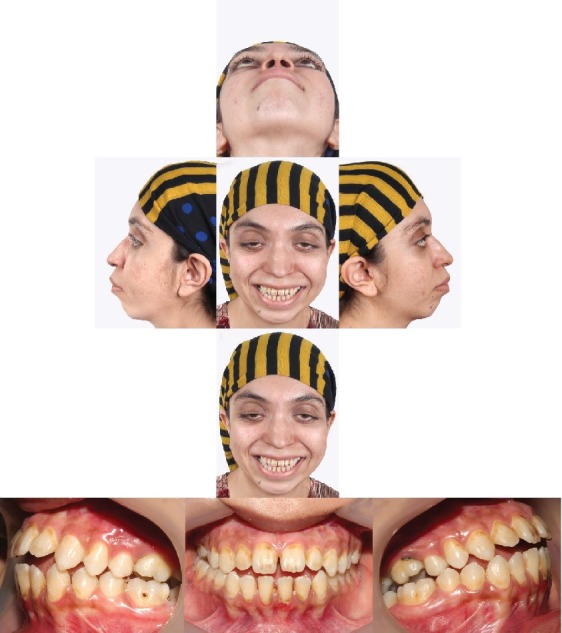
Postreatment patients extraoral and intraoral photographs.

**Figure 6:**
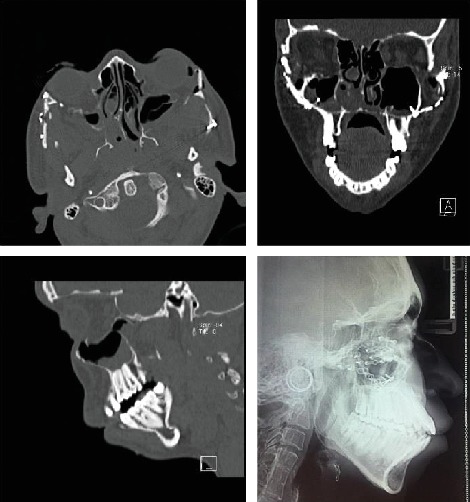
Postreatment axial, sagittal, and coronal computed tomographic views and lateral cephalogram demonstrated correction of orbital depth and exophthalmos.

**Figure 7:**
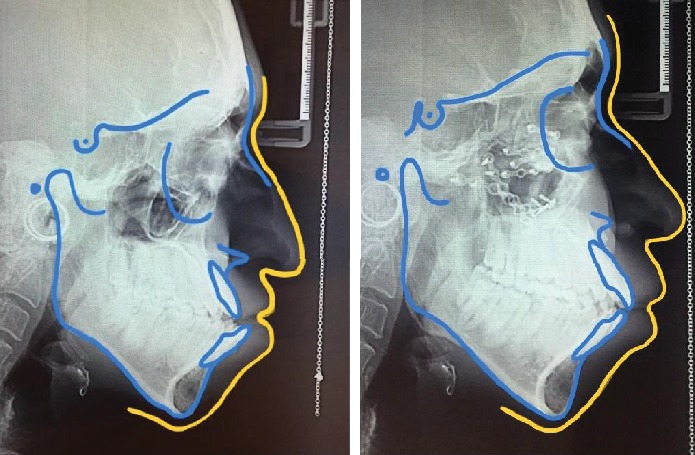
Pre- and posttreatment cephalometric tracings.

Considering the proper contour of the forehead and the supraorbital area and absence of hypoplasia in the nasal unit, modified Le Fort III osteotomy procedure was planned for the patient in order to correct the malar and maxillary deficiency and proptosis. In 1971, Kufner modified the osteotomy plan of Gillis for the first time and made an attempt to advance the midface without disturbing the nasofrontal unit [18]. Kufner's efforts and others resulted in the introduction of the modified Le Fort III osteotomy [19]. In this case, by the modified Le Fort III osteotomy plan, a proper facial profile was achieved. The severity of proptosis and the scleral show were decreased, and the nasal dorsum and nasofrontal areas remained unchanged (Figures 5 and 6). Clockwise rotation of the maxilla resulted in anterior-superior autorotation of the mandible, decreasing the patient's open bite. A class I molar and canine relationship was achieved, and the patent's reverse jet decreased significantly (Figure 5). Comparison of cephalometric analyses before and after surgery indicated the advancement of the maxilla and the lower orbital rim (Table 1 and Figure 7).

Intraoral and extraoral access is used to carry out modified Le Fort III osteotomy. Fariña et al. used a combination of intraoral and transconjunctival incisions in association with canthotomy to gain access to modified Le Fort III osteotomy areas and reported that the advantages of the above approach included less hemorrhage and shorter duration of surgery [20]. In the surgery of the patient reported here, a coronal and intraoral approach was used to gain access to the osteotomy sites. As reported by Tessier, after advancement of the midface, it is necessary to use bone grafts in the fixation areas in order to improve the stability of treatment and achieve proper bone healing [21]. Periorbital approach does not provide enough space for placement of grafts and fixation devices. The advantages of the coronal approach are an inconspicuous scar behind the hairline and the possibility of harvesting a calvarial graft without creating another donor site and its related morbidity. Ridgway studied the severity of lower lid malformation subsequent to periorbital incisions for the reconstruction of traumatic fractures of the face and reported that the highest risk of entropion was related to the transconjunctival approach; the highest risk of ectropion was related to the subciliary approach and the risk of a hypertrophic scar was related to the subtarsal approach [22]. Accessibility and visibility through the osteotomy sits of zygomaticofrontal, infraorbital rim and posterior maxillary walls are highly achieved by coronal approach. Through hypotensive anesthetic condition, proper surgical hemostasis, and oral and maxillofacial surgeons experienced in scalpel incision, the amount of bleeding and surgery time is controllable in the coronal approach.

Some researchers suggest distraction osteogenesis due to need for excessive advancements in patients with craniofacial dysostosis. This technique might result in better stability of the treatment outcomes and greater movement range with no need for bone grafts and no donor site morbidity [23]. On the other hand, this technique requires the patient's full compliance to activate the appliance regularly. The amount and direction of skeletal movements cannot be controlled in contrast to surgical advancements subsequent to osteotomy and the deformity remaining after it might require revision in some cases. The placed distractor appliances should be removed during a second surgery, and the appliances are very expensive in Iran and patients cannot afford it. Distraction treatment was initially considered for the patient reported here. However, due to lack of compliance by the patient and its high cost, it was excluded.

The patient was satisfied with the treatment and reported that it positively affected her social and occupational activities. The patient was followed for six months after treatment. The occlusion which was achieved during surgery had been maintained. The facial profile and the scleral show were favorable and the scar of the coronal approach was hidden behind the hairline, with no alopecia. The patient's visual acuity had improved compared to the preoperative period based on ophthalmologic examinations. In addition, the risk of injuries to the eye globe and corneal keratitis had decreased due to a decrease in proptosis.

## 7. Conclusion

This case report demonstrates that it is possible to treat patients with Crouzon syndrome with severe midface hypoplasia and proptosis by modified Le Fort III osteotomy, with a high success rate, in the absence of presurgical orthodontic treatment.

## Figures and Tables

**Table 1:** Cephalometric analysis.

	Normal value	Pretreatment value	Posttreatment value
A point to N-P	1	-3	2
SNA	82	80	88
U1 to S-N	104	117	120
Nasolabial angle	102	58	65
Occlusal plane angle	17	7	19
Nasofrontal angle	120	120	119
Overjet	2-3	-6	3
Overbite	—	5	1
Molar discrepancy	0	-12	0

## References

[1] Crouzon O. (1912). Dysostose cranio-faciale hereditair. *Bulletins et Mémoires de la Société Médicale des Hôpitaux de Paris*.

[2] Fogh-Andersen P. (1943). Craniofacial dysostosis (Crouzon’s disease) as a dominant hereditary affection. *Nordisk Medicin*.

[3] Cohen M. M., Opitz J. M., Reynolds J. F., Gorlin R. J. (1988). Craniosynostosis update 1987. *American Journal of Medical Genetics*.

[4] Dodge H. W., Wood M. W., Kennedy R. L. (1959). Craniofacial dysostosis: Crouzon's disease. *Pediatrics*.

[5] Franceschetti A. (1968). Cranial dysostosis with pronounced digital impressions (pseudo-crouzon dysostosis). *Congenital Anomalies of the Eye*.

[6] Bertelsen T. (1958). The premature synostosis of the cranial sutures. *Acta Ophthalmologica Supplement*.

[7] Jabs E. W., Li X., Scott A. F. (1994). Jackson-Weiss and Crouzon syndromes are allelic with mutations in fibroblast growth factor receptor 2. *Nature Genetics*.

[8] Bowling E. L., Burstein F. D. (2006). Crouzon syndrome. *Optometry - Journal of the American Optometric Association*.

[9] Järund M., Laurcitzen C. (1996). Craniofacial dysostosis: airway obstruction and craniofacial surgery. *Scandinavian Journal of Plastic and Reconstructive Surgery and Hand Surgery*.

[10] Pinkerton O., Pinkerton F. (1952). Hereditary craniofacial dysplasia. *American Journal of Ophthalmology*.

[11] Vu D. D., Tiwana P. S. (2016). Le Fort III and Le Fort II osteotomies. *Atlas of the Oral and Maxillofacial Surgery Clinics*.

[12] Kapp-Simon K. A., Simon D. J., Kristovich S. (1992). Self-perception, social skills, adjustment, and inhibition in young adolescents with craniofacial anomalies. *The Cleft Palate-Craniofacial Journal*.

[13] Pertschuk M., Whitaker L. (1982). Social and psychological effects of craniofacial deformity and surgical reconstruction. *Clinics in Plastic Surgery*.

[14] Whitaker L. A., Munro I. R., Salyer K. E., Jackson I. T., Ortiz-Monasterio F., Marchac D. (1979). Combined report of problems and complications in 793 craniofacial operations. *Plastic and Reconstructive Surgery*.

[15] Fort R. E. N. É., Tessier P. (1972). Experimental study of fractures of the upper jaw. *Plastic and Reconstructive Surgery*.

[16] Gillies H., Harrison S. H. (1950). Operative correction by osteotomy of recessed malar maxillary compound in a case of oxycephaly. *Plastic and Reconstructive Surgery*.

[17] Tessier P. (1967). Osteotomies totales de la face. Syndrome de Crouzon, syndrome d'Apert: oxycephalies, scaphocephalies, turricephalies. *Annales de Chirurgie Plastique*.

[18] Kufner J. (1971). Four-year experience with major maxillary osteotomy for retrusion. *Journal of oral surgery (American Dental Association : 1965)*.

[19] Epker B. N., Wolford L. M. (1975). Middle-third facial osteotomies: their use in the correction of acquired and developmental dentofacial and craniofacial deformities. *Journal of oral surgery (American Dental Association: 1965)*.

[20] Fariña R., Valladares S., Raposo A., Silva F. (2018). Modified Le Fort III osteotomy: a simple solution to severe midfacial hypoplasia. *Journal of Cranio-Maxillofacial Surgery*.

[21] Tessier P. (1971). The definitive plastic surgical treatment of the severe facial deformities of craniofacial dysostosis: Crouzon's and Apert's diseases. *Plastic and Reconstructive Surgery*.

[22] Ridgway E. B., Chen C., Colakoglu S., Gautam S., Lee B. T. (2009). The incidence of lower eyelid malposition after facial fracture repair: a retrospective study and meta-analysis comparing subtarsal, subciliary, and transconjunctival incisions. *Plastic and Reconstructive Surgery*.

[23] Nout E., Wolvius E., van Adrichem L., Ongkosuwito E., van der Wal K. (2006). Complications in maxillary distraction using the RED II device: a retrospective analysis of 21 patients. *International Journal of Oral and Maxillofacial Surgery*.

